# Phase I study of DFP-11207, a novel oral fluoropyrimidine with reasonable AUC and low C_max_ and improved tolerability, in patients with solid tumors

**DOI:** 10.1007/s10637-020-00939-w

**Published:** 2020-05-06

**Authors:** Jaffer A. Ajani, Milind Javle, Cathy Eng, David Fogelman, Jackie Smith, Barry Anderson, Chun Zhang, Kenzo Iizuka

**Affiliations:** 1 Department of Gastrointestinal Medical Oncology, Division of Cancer Medicine, The University of Texas MD Anderson Cancer Center, Houston, TX USA; 2 Theradex, Princeton, NJ USA; 3 Delta-Fly Pharma, Inc., Tokushima, Japan

**Keywords:** 5-FU derivative, Chemotherapy, Solid tumor, Dihydropyrimidine dehydrogenase

## Abstract

5-fluorouracil (5-FU) and 5-FU derivatives, such as capecitabine, UFT, and S-1, are the mainstay of chemotherapy treatment for gastrointestinal cancers, and other solid tumors. Compared with other cytotoxic chemotherapies, these drugs generally have a favorable safety profile, but hematologic and gastrointestinal toxicities remain common. DFP-11207 is a novel oral cytotoxic agent that combines a 5-FU pro-drug with a reversible DPD inhibitor and a potent inhibitor of OPRT, resulting in enhanced pharmacological activity of 5-FU with decreased gastrointestinal and myelosuppressive toxicities. In this Phase I study (NCT02171221), DFP-11207 was administered orally daily, in doses escalating from 40 mg/m^2^/day to 400 mg/m^2^/day in patients with esophageal, colorectal, gastric, pancreatic or gallbladder cancer (*n* = 23). It was determined that DFP-11207 at the dose of 330 mg/m^2^/day administered every 12 hours was well-tolerated with mild myelosuppressive and gastrointestinal toxicities. The pharmacokinetic analysis determined that the 5-FU levels were in the therapeutic range at this dose. In addition, fasted or fed states had no influence on the 5-FU levels (patients serving as their own controls). Among 21 efficacy evaluable patients, 7 patients had stable disease (33.3%), of which two had prolonged stable disease of >6 months duration. DFP-11207 can be explored as monotherapy or easily substitute 5-FU, capecitabine, or S-1 in combination regimens.

## Introduction

5-Fluorouracil (5-FU), an antimetabolite, was introduced by Heidelberger et al. [1] in 1957, and has since been widely used as a single agent or in combination with other drugs [2–4] mainly in localized or metastatic gastrointestinal cancers and breast cancer. Clinical response and toxicity of 5-FU are remarkably influenced by its dosing schedule and a prolonged exposure by continuous infusion of 5-FU has been found to increase tumor response rates [5–12].

Seeking the efficacy advantage of a continuous infusion 5-FU schedule and in order to enhance patient compliance [13, 14], several oral 5-FU derivatives such as capecitabine [15], tegafur-uracil (UFT) [16] and tegafur-gimeracil-oteracil (S-1) [17] have been developed. Available oral fluoropyrimidines, although considered efficacious have toxicities that remain an ongoing concern. The primary dose-limiting toxicity of capecitabine, UFT, and S-1 has been shown to be hand-foot syndrome [18], gastrointestinal toxicity [19] and hematological toxicity [20], respectively.

A second generation oral fluoropyrimidine, UFT, that combines tegafur [5-fluoro-1-(tetrahydro-2-furanyl)-2,4(1H,3H)-pyrimidinedione] with the DPD inhibitor uracil, has shown significantly less hematological and gastrointestinal toxicity [21–23]., A fourth generation oral fluoropyrimidine, S-1, that combines tegafur with 5-chloro-2,4-dihydroxypyridine (CDHP; a reversible inhibitor of 5-FU degradation [24–27]), and potassium oxonate [potassium 1,3,5-triazine 2,4(1H,3H)-dione-6-carboxylate] that inhibits orotate phosphoribosyl-transferase, the enzyme that phosphorylates 5-FU in the gastrointestinal tract, demonstrated further reduction in gastrointestinal toxicity of 5-FU [17, 24, 27–33].

Tumor-selective cytotoxicity of S-1 was confirmed in two Phase I [24, 33] and four Phase II trials in patients with advanced gastric or colorectal cancer [20, 34–36], which led to approval of S-1 for patients with advanced gastric cancer in Japan [34]. In all of the above studies hematological toxicity was predominant, with only a few or no cases of ≥ Grade 3 gastrointestinal toxicity. Notably, hematological toxicities predominated in the studies conducted in Japan, and gastrointestinal toxicity was the DLT in Europe and the United States [20, 24, 25, 33–37], likely due to a lower expression of the cytochrome P4502A6 (CYP2A6) isozyme in Japanese individuals [24, 25, 34, 35, 38–42].

Interindividual and intraindividual variations in plasma 5-FU concentrations are mainly caused by differing levels of dihydropyrimidine dehydrogenase (DPD), the primary catabolic enzyme of 5-FU [43–45]. Deficiency of DPD is associated with severe hematologic and gastrointestinal toxicity after 5-FU administration [46]. Inhibition or inactivation of DPD has emerged as a potential strategy to reduce the pharmacokinetic variability and improve the efficacy of 5-FU [12, 47].

To minimize the 5-FU-induced toxicities without compromising its antitumor activity, Delta-Fly Pharma, Inc. has developed DFP-11207, a novel cytotoxic agent that combines a 5-FU pro-drug (1-ethoxymethyl-5-fluorouracil; EM-FU) [26] with a reversible DPD inhibitor CDHP [48] and a potent inhibitor of orotate phosphoribosyl transferase (citrazinic acid; CTA). This combination results in enhanced pharmacological activity of 5-FU, with lower C_max_ and AUC values but longer T_max_ and T_1/2_ values of 5-FU, respectively than S-1, which suggests that DFP-11207 may be superior in preventing the 5-FU-induced severe hematological and gastrointestinal toxicities. CTA, that is mainly retained in gastrointestinal tract cells, protects the gastrointestinal tract from injury by inhibiting 5-FU phosphorylation.

DFP-11207’s self-controlled toxicity profile may allow this molecule to improve the tolerability and efficacy of 5-FU-based treatment for cancer patients as a monotherapy or in combination therapy [49].

## Materials and methods

### Study design

This Phase I, open-label, single arm, single-center, dose escalation, safety, tolerability, and pharmacokinetic (PK) study of DFP-11207 in patients with advanced solid tumors (NCT02171221) was sponsored by Delta-Fly Pharma, Inc. (Tokushima, Japan).

The primary objective of this study was to determine the maximum tolerated dose (MTD), the recommended Phase II dose (RP2D) and the dose-limiting toxicity (DLT) of DFP-11207 in patients with advanced solid tumors, and to assess pharmacokinetic (PK) profiles of DFP-11207 under fed and fasted conditions. Secondary objectives were to perform PK analysis of DFP-11207 and to assess the antitumor activity of DFP-11207 in patients with advanced solid tumors.

### Eligibility

Eligible patients were males or females of at least 18 years of age, with solid tumors refractory by standard therapies or for which conventional chemotherapy was not reliably effective or no effective therapy was available. Patients must have had adequate bone marrow function as defined by absolute neutrophil count of ≥1.5 × 10 ^9^/L and platelets of ≥100 × 10 ^9^/L. Adequate liver and kidney function were required. Patient who had current malignancies of another type, patients after extensive prior radiotherapy, or prior bone marrow/stem cell transplantation, and patients with clinically evident CNS metastases or leptomeningeal disease were not eligible for the study. Prior exposure to chemotherapy, immunotherapy, radiotherapy or any other investigational therapy within 4 weeks was not permitted. Patients with cardiac dysfunction and known bleeding disorder were excluded.

### Treatment

In the Phase I Study, patients received DFP-11207 capsules orally, daily in 28-day cycles. Patients in the 40 to 250 mg/m^2^/day cohorts received once-daily DFP-11207 dosing. For patient compliance, the DFP-11207 dosing schedule was changed at doses of 330 mg/m^2^/day (1 patient) or 440 mg/m^2^/day (4 patients) to every 12 hours on all treatment days except on Cycle 1, Days 1 and 29, when DFP-11207 was administered as a single dose for PK sampling purposes and later for the subsequent 5 patients in the 330 mg/m^2^/day cohort, DFP-11207 dosing schedule was changed to every 12 hours on all treatment days. Subsequently, a Food Effect Study was added, and 6 patients were treated with DFP-11207 at the dose of 600 mg/day administered as 300 mg every 12 hours.

### Criteria for evaluation

#### Safety

Safety data including laboratory parameters, vital signs, and adverse events were collected for all patients. All patients who received any amount of DFP-11207 were included in the safety analysis. Safety parameters evaluated include adverse events, vital signs, and clinical laboratory results. Adverse events were classified according to the National Cancer Institute (NCI) Common Terminology Criteria for Adverse Events (CTCAE) Version 4.0.

#### Efficacy

Although response was not the primary endpoint of this study, patients with measurable disease were assessed using RECIST version 1.1, where possible after every 2 cycles. For patients with less than 2 cycles of study therapy, if there was clear evidence of clinical progression then they were considered eligible for the efficacy evaluation.

#### Plasma pharmacokinetics, phase I study

Following oral administration of DFP-11207, blood samples were collected during Cycle 1 on Day 1 at 0 hour (pre-dose), 1, 2, 4, 8, 12, 24, and 48 hours after the study drug administration and thereafter, on day 15 pre-dose (added per amendment 2 to monitor every 12 hours dosing) and day 29, pre-dose and 4 hours after DFP-11207 administration. DFP-11207 metabolites: 5-fluorouracil (5-FU), 1-ethoxymethyl-5-fluorouracil (EM-FU), 5-chloro-2,4-dihydroxypyridine (CDHP), and citrazinic acid (CTA) were then measured.

#### Plasma pharmacokinetics, food effect study

Following oral administration of DFP-11207, blood samples were collected during Cycle 1 (fed or fasted study days) on Day 1 at 0 hour (pre-dose), and 4, 10, 24 and 48 hours after the study drug administration, Day 8 at 2 hours after the morning DFP-11207 administration, Day 14 at 2, 10 and 24 hours after DFP-11207 administration, Day 16 at 0 hour (pre-dose), and 4, 10 and 24 hours after DFP-11207 administration, Day 18 at pre-dose, Day 23 at 2 hours after the morning DFP-11207 administration, Day 29 at 2, 10 and 24 houras after DFP-11207 administration, and pre-dose on Cycle 2 Day 1.

For both the Phase I Study and the Food Effect Study, whole blood (5 mL) was collected in chilled heparin collection tubes to harvest plasma. High performance liquid chromatography with tandem mass spectrometry (LC-MS/MS) was used to determine plasma and urine concentrations of DFP-11207 metabolites 5-FU, EM-FU, CDHP, and CTA.

#### Urine pharmacokinetics, phase I study

Urine samples (8 mL) were also collected and pooled at pre-dose (−12 to 0 hour) and after the start of DFP-11207 treatment at: 0 to 12 hours, 12 to 24 hours, 24 to 36 hours, and 36 to 48 hours. DFP-11207 metabolites were then measured.

### Statistical approach

The primary endpoint of this study was to assess the toxicity of DFP-11207 by determining the dose level at which DLTs were observed. AEs were arranged by decreasing frequency of AEs. Laboratory data was graded according to NCI CTCAE (Version 4.0) and tabulated based on maximum grade. AEs were coded using the Medical Dictionary for Drug Regulatory Activities (MedDRA^®^) Version 17.0.

Secondary endpoints of this study were to examine the efficacy and PKs of DFP-11207. For efficacy analysis, overall response was assessed using RECIST version 1.1. The objective antitumor response rate [complete response (CR) or partial response (PR)] was calculated as the proportion of patients with responsive disease (CR + PR) and 95% confidence interval for response was calculated for the median time. Duration of response and time to tumor progression were evaluated using Life Table methods. Life Table estimates were calculated using Kaplan-Meier methodology.

Plasma concentration and time data of DFP-11207 metabolites (5-FU, EM-FU, CDHP and CTA) in the Food Effect Study were determined using non-compartmental methods (WinNonLin^®^). PK parameters to be calculated included AUC extrapolated to infinity (AUC_inf_), peak concentration (C_max_), time at C_max_ (T_max_) and elimination half-life (T_1/2_). Statistical analysis of PK parameters was to be performed to compare each fed condition to the fasted condition by using analysis of variance which were compared by using the Wilcoxon matched-pairs test.

## Results

### Patient demographics

Among 23 patients enrolled in the study, 13 patients (56.5%) were men and 10 patients (43.5%) were women. The median patient age was 59 years (overall age range: 36 to 86 years) and median body surface area (BSA) was 1.94 m^2^ (range: 1.57 to 2.58). Seventeen patients (73.9%) were White, 3 patients (13.0%) were Black or African American, 2 patients (8.7%) were Asian and 1 patient (4.3%) was of other race. At baseline, 5 patients (21.7%) had Eastern Cooperative Oncology Group (ECOG) performance status of 0 and 18 patients (78.3%) had ECOG performance status of 1.

### Baseline disease characteristics

The majority of patients (19 patients; 82.6%) had histopathology diagnosis of adenocarcinoma not otherwise specified (NOS); 4 patients (17.4%) had carcinoma NOS as the predominant histopathology with primary tumors sites of esophagus or rectum (both 6 patients each), large intestine (4 patients), pancreas or stomach (3 patients each) and gallbladder and extrahepatic bile duct (1 patient) (see Tables 1, 2 and 3).

**Table 1:** Prior cancer therapy

Intent-to-Treat (N = 23)										
	DFP-11207 Dose Cohort (mg/m^2^/day)									
Prior therapy^1,2^	40	80	110	140	190	250	330	440	600 mg/day	Overall
Number of patients	1	2	1	1	1	1	6	4	6	23
Number of patients with:										
Prior chemotherapy	1 (100.0%)	2 (100.0%)	1 (100.0%)	1 (100.0%)	1 (100.0%)	1 (100.0%)	6 (100.0%)	4 (100.0%)	6 (100.0%)	23 (100.0%)
Prior immunotherapy	0	1 (50.0%)	0	0	0	0	0	0	1 (16.7%)	2 (8.7%)
Other prior therapy	1 (100.0%)	2 (100.0%)	1 (100.0%)	1 (100.0%)	0	1 (100.0%)	4 (66.7%)	2 (50.0%)	1 (16.7%)	13 (56.5%)
Prior radiation therapy	0	2 (100.0%)	1 (100.0%)	0	1 (100.0%)	1 (100.0%)	2 (33.3%)	3 (75.0%)	3 (50.0%)	13 (56.5%)
Prior surgery	1 (100.0%)	2 (100.0%)	1 (100.0%)	1 (100.0%)	1 (100.0%)	1 (100.0%)	6 (100.0%)	4 (100.0%)	6 (100.0%)	23 (100.0%)

^1^Patients may be counted in more than one prior therapy category

^2^ Number of patients used as denominator to calculate percentages

**Table 2:** Baseline disease characteristics

Intent-to-Treat (N = 23)										
	DFP-11207 Dose Cohort (mg/m^2^/day)									
Disease characteristics	40	80	110	140	190	250	330	440	600 mg/day	Overall
Number of Patients	1	2	1	1	1	1	6	4	6	23
Histopathology^1^										
Adenocarcinoma, NOS	0	2 (100.0%)	0	1 (100.0%)	0	1 (100.0%)	6 (100.0%)	3 (75.0%)	6 (100.0%)	19 (82.6%)
Carcinoma, NOS	1 (100.0%)	0	1 (100.0%)	0	1 (100.0%)	0	0	1 (25.0%)	0	4 (17.4%)
Primary site^1^										
Esophagus	0	1 (50.0%)	0	0	0	0	1 (16.7%)	2 (50.0%)	2 (33.3%)	6 (26.1%)
Rectum	0	0	0	0	1 (100.0%)	0	1 (16.7%)	1 (25.0%)	3 (50.0%)	6 (26.1%)
Large intestine, (excl. appendix)	1 (100.0%)	0	0	0	0	1 (100.0%)	2 (33.3%)	0	0	4 (17.4%)
Pancreas	0	1 (50.0%)	0	1 (100.0%)	0	0	1 (16.7%)	0	0	3 (13.0%)
Stomach	0	0	0	0	0	0	1 (16.7%)	1 (25.0%)	1 (16.7%)	3 (13.0%)
Gallbladder & extrahepatic bile ducts	0	0	1 (100.0%)	0	0	0	0	0	0	1 (4.3%)

^1^Number of treated patients used as denominator to calculate percentages

**Table 3:** Duration of disease at baseline

Intent-to-Treat (N = 23)										
	DFP-11207 Dose Cohort (mg/m^2^/day)									
Duration of disease (months)	40	80	110	140	190	250	330	440	600 mg/day	Overall
N	1	2	1	1	1	1	6	4	6	23
Mean	13.24	23.44	29.34	20.99	19.88	62.82	51.53	15.60	38.43	34.58
Standard deviation		0.813					40.595	7.891	33.519	29.564
Median	13.24	23.44	29.34	20.99	19.88	62.82	41.89	16.92	30.85	23.59
Minimum	13.2	22.9	29.3	21.0	19.9	62.8	10.0	5.0	9.9	5.0
Maximum	13.2	24.0	29.3	21.0	19.9	62.8	115.3	23.6	103.1	115.3

All 23 patients had prior surgery and chemotherapy; 13 patients (56.5%) had prior radiation therapy; 2 patients (8.7%) had prior immunotherapy. In addition, 13 patients (56.5%) had other prior therapy (see Table 1).

#### Treatment

**Fig. 1:**
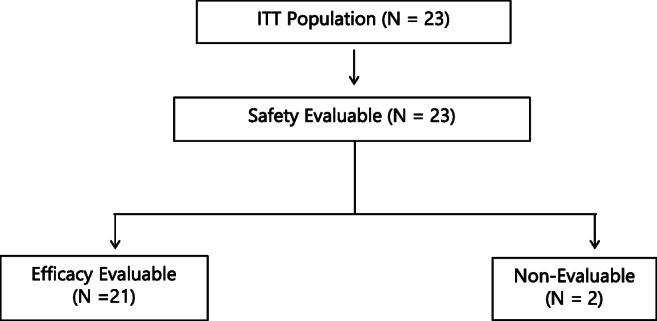
Disposition of patients (*N* = 23)

During the Phase I Study, DFP-11207 dose escalation progressed in accelerated single patient cohorts from 40 mg/m^2^/day up to 330 mg/m^2^/day; no Grade 2 or greater DFP-11207-related AEs were observed during Cycle 1 of dosing at these doses (Fig. 1). At 440 mg/m^2^/day, the first patient enrolled in the dose cohort experienced DFP-11207-related AEs of Grade 3 dehydration and mucosal inflammation and Grade 4 febrile neutropenia. Per protocol, the 440 mg/m^2^/day cohort was expanded to 3 patients. The 3rd patient enrolled in the 440 mg/m^2^/day cohort experienced a Grade 4 febrile neutropenia. As per the Safety Review Committee assessment, Grade 4 febrile neutropenia was considered a DLT. Thus, dose escalation was stopped, and 440 mg/m^2^/day was declared to be the maximum administered dose (MAD). Five additional patients were treated every 12 hours at 330 mg/m^2^/day, the dose below 440 mg/m^2^/day. In total, during the Phase I Study, 6 patients - 1 during the dose escalation phase and 5 during the MTD confirmation phase - were treated at 330 mg/m^2^/day. No DLTs were reported at the dose of 330 mg/m^2^/day. Therefore, 330 mg/m^2^/day administered every 12 hours was confirmed as the MTD, which is also the RP2D. In the Food Effect Study, all 6 patients were treated with DFP-11207 at the dose of 600 mg/day administered as 300 mg every 12 hours; no DLTs were reported.

#### Adverse events

A summary of the ≥ Grade 3 treatment emergent adverse events (TEAEs) that were reported during the study is presented in Table 4. All 23 safety evaluable patients who received DFP-11207 treatment (17 patients in the Phase I Study, 6 patients in the Food Effect Study) experienced at least 1 TEAE. A total of 16 patients (69.6%) reported TEAEs related to DFP-11207. The incidence of ≥ Grade 3 drug-related TEAEs was 13.0% (3/23 patients; all in the Phase I Study including, 1/6 patients in the 330 mg/m^2^/day cohort and 2/4 patients in the 440 mg/m^2^/day cohort). The most frequently reported (≥ 10% of patients) ≥ Grade 3 events were dysphagia (13.0%), dehydration (13.0%), and failure to thrive (13.0%). The most common ≥ Grade 3 drug-related hematologic AE reported in 440 mg/m^2^/day cohort was febrile neutropenia (2 patients; 8.7%); anemia (1 patient; 4.3%) and pancytopenia (1 patient; 4.3%). Notably, no patient experienced any ≥ Grade 3 drug-related thrombocytopenia. Two patients (8.7%) in the Phase I Study had drug-related SAEs. There was no death related to DFP-11207 treatment. DFP-11207 dose interruption due to TEAEs occurred in 34.8% of patients (8/23 patients; all in the Phase I Study, including 1/2 patients in the 80 mg/m^2^/day cohort:, 1/1 patient in the 250 mg/m^2^/day cohort, 2/6 patients in the 330 mg/m^2^/day cohort, and 4/4 patients in the 440 mg/m^2^/day cohort), indicating DFP-11207 was well-tolerated at the RP2D level of 330 mg/m^2^/day every 12 hours.

**Table 4:** Summary of ≥ grade 3 treatment-emergent adverse events by system organ class and preferred term

MedDRA System Organ Class MedDRA Preferred Term	DFP-11207 Dose cohort (mg/m^2^/day)		
	330	440	600 mg/day
Number of patients	6	4	6
Number of Patients with Any Grade 3 or Greater, Drug-Related, TEAEs	1 (16.7%)	2 (50.0%)	0
Blood and lymphatic system disorders	0	2 (50.0%)	0
Febrile neutropenia	0	2 (50.0%)	0
Anemia	0	1 (25.0%)	0
Pancytopenia	0	1 (25.0%)	0
Gastrointestinal disorders	1 (16.7%)	0	0
Vomiting	1 (16.7%)	0	0
General disorders and administration site conditions	0	1 (25.0%)	0
Mucosal inflammation	0	1 (25.0%)	0
Metabolism and nutrition disorders	0	1 (25.0%)	0
Dehydration	0	1 (25.0%)	0

Overall, the most frequently reported drug-related TEAEs (≥ 10% of patients) were fatigue (47.8%), nausea (47.8%), decreased appetite (39.1%), diarrhea (26.1%), vomiting (21.7%), anemia (13.0%), dysgeusia (13.0%), mucosal inflammation (13.0%) and palmar-plantar erythrodysesthesia syndrome (13.0%). In the Phase I Study, at the MTD/RP2D (330 mg/m^2^/day), drug-related TEAEs reported were decreased appetite (4 of 6 patients [4/6]), fatigue (4/6), nausea (4/6), vomiting (2/6), diarrhea (1/6), hematemesis (1/6), lacrimation increase (1/6), microcytic anemia (1/6), nail discoloration (1/6), ocular hyperemia (1/6), palmar-plantar erythrodysesthesia syndrome (1/6), pyrexia (1/6), and stomatitis (1/6), of which vomiting was the only ≥ Grade 3 drug-related TEAE (1 patient). In the Food Effect Study, the DFP-11207-related TEAEs reported were fatigue (3 patients; 50%), diarrhea (2 patients; 33.3%), and constipation, decreased appetite, insomnia, mucosal inflammation, paresthesia, urinary tract infection, vomiting (1 patient each; 16.7%). No ≥ Grade 3 TEAEs were reported in the Food Effect Study.

#### Pharmacokinetics

The PK data at 330 and 440 mg/m^2^/day indicate that dose-dependent plasma 5-FU concentrations ranged from 4.01 to 25.5 ng/mL and 3.39 to 13.0 ng/mL on Cycle 1, Day 2 and 5.27 to 23.5 ng/mL and 23 to 45.3 ng/mL on Cycle 1, Day 29 (pre-dose), respectively. Plasma EM-FU concentrations at 330 and 440 mg/m^2^/day ranged from 559 to 1380 ng/mL and 948 to 1770 ng/mL on Cycle 1, Day 2 and 2510 to 5120 ng/mL and 3440 to 5900 ng/mL on Cycle 1, Day 29 (pre-dose), respectively. Plasma 5-FU and EM-FU concentrations on Cycle 1, Day 15 (pre-dose) at the dose of 330 mg/m^2^/day ranged from 9.21 to 119 ng/mL and 2760 to 8750 ng/mL, respectively (Table 5). Day 29 plasma concentrations for all analytes were higher than Day 1 plasma concentrations at the same timepoint, suggesting accumulation with repeat daily dosing of DFP-11207. Overall, the PK data suggest that DFP-11207 at these dose levels maintain blood concentrations of 5-FU of approximately 20 ng/mL throughout the dose cycle in patients with advanced solid tumors. In contrast, the urinary excretion of 5-FU, EM-FU, CDHP and CTA during Cycle 1 0–48 hours after the DFP-11207 dose of 330 mg/m^2^/day accounted for 0.12, 0.71, 1.98 and 0.07%, respectively.

**Table 5:** Summary of Plasma PK Parameters by Analyte Following Dosing of 330 and 440 mg/m^2^ Oral DFP-11207 on Day 1 in Patients with Solid Tumors

Dose level (mg/m^2^)	Parameter (unit)	Statistic	EM-FU	5-FU	CDHP	CTA
330	T_max_ (h)	N; Median (Min, Max)	6; 48 (24, 48)	6; 24 (24, 48)	6; 24 (8.0, 48)	5; 8.0 (2.0, 48)
	C_max_ (ng /mL)	N; Mean (SD)	6; 1310 (370)	6; 9.65 (8.25)	6; 90.4 (39.7)	5; 9.81 (5.68)
	AUC_last_ (ng·h/mL)	N; Mean (SD)	6; 39,900 (8950)	1; 220	6; 2410 (1080)	4; 254 (134)
440	T_max_ (h)	N; Median (Min, Max)	4; 24 (24, 48)	3; 12 (8.0, 48)	4; 26 (2.0, 48)	3; 4.0 (4.0, 8.0)
	C_max_ (ng /mL)	N; Mean (SD)	4; 1400 (595)	3; 8.75 (5.24)	4; 93.6 (60.5)	3; 9.72 (6.53)
	AUC_last_ (ng·h/mL)	N; Mean (SD)	4; 49,400 (18200)	3; 311 (214)	4; 2810 (2150)	2; 388 (237)

**Fig. 2:**
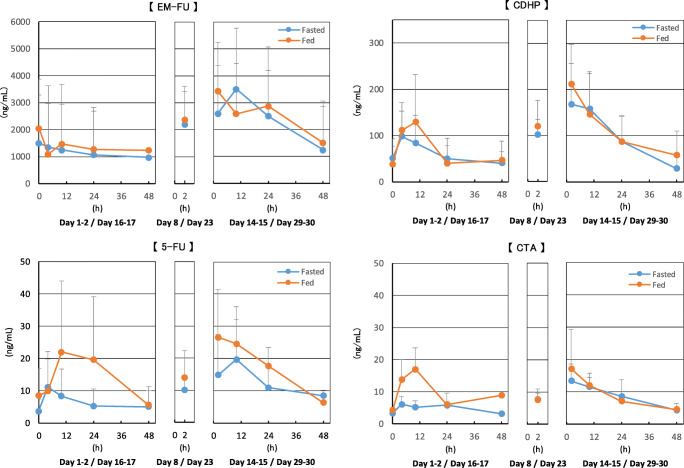
PK Parameters in food effect study (*N* = 6)

Among 6 patients receiving a DFP-11207 dose of 300 mg twice daily both with food or without food the PK results determined that EM-FU and CDHP were detectable in plasma at all time points of assessment during fed and fasted dosing. While 5-FU and CTA plasma levels had more variability in patients, there were no clear differences in C_max_ or AUC_last_ between DFP-11207 taken under fasted and fed conditions (Table 6). An approximately 1.5- to 3-fold increase in AUC_last_ was observed following the steady state dose compared to the initial dose. Based on these results, DFP-11207 bioavailability does not appear to differ substantially whether patients are administered DFP-11207 in a fed or fasted state (Fig. 2).

**Table 6:** Summary of PK parameters in food effect study

Period dose	State	Parameter (unit)	Statistic	EM-FU	5-FU	CDHP	CTA
Initial dose	Fasted	T_max_ (h)	N; Median (Min, Max)	6; 36 (4.0, 48)	5; 10 (4.0, 48)	6; 7.0 (4.0, 48)	4; 4.0 (4.0, 24)
		C_max_ (ng /mL)	N; Mean (SD)	6;1400 (1260)	5; 7.36 (4.69)	6; 91.9 (46.9)	4; 7.04 (2.53)
		AUC_last_ (ng·h/mL)	N; Mean (SD)	6; 49,800 (53800)	1; 275	6; 2650 (1810)	2; 174 (53.9)
Initial dose	Fed	T_max_ (h)	N; Median (Min, Max)	5; 10 (4.0, 48)	4; 10 (10, 48)	5; 4.0 (4.0, 10)	4; 10 (4.0, 48)
		C_max_ (ng /mL)	N; Mean (SD)	5; 1580 (1620)	4; 22.9 (22.3)	5; 154 (88.2)	4; 17.3 (5.97)
		AUC_last_ (ng·h/mL)	N; Mean (SD)	5; 56,500 (70600)	2; 655 (600)	5; 3000 (2120)	1; 376
Steady state dose	Fasted	T_max_ (h)	N; Median (Min, Max)	6; 10 (2.0, 24)	6; 6.0 (2.0, 24)	6; 2.0 (2.0, 24)	6; 2.0 (2.0, 24)
		C_max_ (ng /mL)	N; Mean (SD)	6; 2940 (2000)	6; 16.7 (14.9)	6; 160 (89.7)	6; 11.7 (6.15)
		AUC_last_ (ng·h/mL)	N; Mean (SD)	5; 94,500 (69600)	3; 535 (360)	5; 3800 (1780)	3; 328 (101)
Steady state dose	Fed	T_max_ (h)	N; Median (Min, Max)	6; 10 (2.0, 24)	5; 2.0 (2.0, 10)	6; 2.0 (2.0, 10)	5; 2.0 (2.0, 2.0)
		C_max_ (ng /mL)	N; Mean (SD)	6; 3180 (2080)	5; 27.6 (14.5)	6; 189 (94.9)	5; 17.2 (12.1)
		AUC_last_ (ng·h/mL)	N; Mean (SD)	5; 10,2000 (84000)	2; 809 (214)	5; 4660 (3090)	2; 371 (43.8)
Ratio steady state dose: initial dose	Fasted	AR AUC_last_	N; Mean (SD)	5; 2.48 (2.22)	1; 2.36	5; 1.69 (1.48)	2; 1.68 (0.17)
	Fed	AR AUC_last_	N; Mean (SD)	4; 3.04 (1.59)	1; 2.85	4; 1.64 (0.62)	0; Not Applicable

#### Efficacy

Among 21 efficacy evaluable patients, 7 patients had stable disease (33.3%; 2 patients in the 330 mg/m^2^/day cohort and 1 patient each in the 250 and 440 mg/m^2^/day cohorts of the Phase I Study, and 3 patients in the Food Effect Study treated at the dose of 600 mg/day); no patients achieved CR or PR (Table 7). Among the 7 patients with stable disease, 2 patients in the Phase I Study had prolonged stable disease of over 6 months duration, 238 days (67-year-old male with colon cancer in the 330 mg/m^2^/day cohort) and 341 days (37-year-old female with colon cancer in the 250 mg/m^2^/day cohort) respectively, suggesting clinical benefit for DFP-11207.

**Table 7:** Best overall response summary

Efficacy evaluable (*N* = 21)										
	DFP-11207 Dose Cohort (mg/m^2^/day)									
Best overall response^1,2,3^	40	80	110	140	190	250	330	440	600 mg/day	Overall
Number of patients	1	2	1	0	1	1	6	3	6	21
Stable disease (SD)	0	0	0	0	0	1 (100.0%)	2 (33.3%)	1 (33.3%)	3 (50.0%)	7 (33.3%)
Progressive disease (PD)	1 (100.0%)	0	1 (100.0%)	0	1 (100.0%)	0	3 (50.0%)	0	2 (33.3%)	8 (38.1%)
Not evaluable^4^	0	2 (100.0%)	0	0	0	0	1 (16.7%)	2 (66.7%)	1 (16.7%)	6 (28.6%)
Overall Response(CR + PR)	0 (0.0%)	0 (0.0%)	0 (0.0%)	0	0 (0.0%)	0 (0.0%)	0 (0.0%)	0 (0.0%)	0 (0.0%)	0 (0.0%)
Lower 95% confidence limit	(0.0%)	(0.0%)	(0.0%)	(0.0%)	(0.0%)	(0.0%)	(0.0%)	(0.0%)	(0.0%)	(0.0%)
Upper 95% confidence limit	(97.5%)	(84.2%)	(97.5%)	0	(97.5%)	(97.5%)	(45.9%)	(70.8%)	(45.9%)	16.1%)

^1^Overall Response based on patients with either a Complete Response (CR) or Partial Response (PR)

^2^ Number of Patients used as denominator to calculate percentages

^3^ Clopper-Pearson method used for the calculation of the 95% confidence interval

^4^ Includes patients with no post-baseline tumor assessments and/or symptomatic deterioration and/or death due to any cause

## Discussion

The patient experience in this Phase I dose escalation study of DFP-11207 indicates successful implementation of a two-pronged strategy to control the toxicity of orally administered 5-FU while maintaining effective circulating levels of 5-FU. CTA is mainly retained in the gastrointestinal tract cells where it inhibits 5-FU phosphorylation, thus protecting the gastrointestinal tract from injury as the 5-FU is absorbed into the circulation. Secondly, CDHP reversibly inhibits DPD to delay the enzymatic degradation of 5-FU in the circulation while associated with the degradation of 5-FU prodrug, resulting in a prolonged systemic 5-FU exposure profile with lower C_max_ and similar AUC compared to S-1. Owing to the concomitant presence of these DFP-11207 components, treatment at the DFP-11207 dose of 330 mg/m^2^/day administered orally every 12 hours produced a steady state circulating 5-FU level of 5.27–23.5 ng/mL (C_max_ < 25 ng/mL) and was well tolerated without any significant myelosuppression or gastrointestinal toxicity in advanced solid tumor patients, while patients treated at 440 mg/m^2^/day had steady state circulating 5-FU levels of 23–45.3 ng/mL (C_max_ 49.7–54.7 ng/mL) associated with severe myelosuppression and moderate mucosal inflammation, fatigue and palmar-plantar erythrodysesthesia.

The food-effect study demonstrates maintenance of bioavailability when DFP-11207 is administered as a twice-daily 300 mg flat-dose with or without food (mean steady state levels of 27.6 and 16.7 ng/mL, mean AUC of 809 and 535 ng·h/mL, respectively) and no significant myelosuppressive or gastrointestinal adverse events. These results are in contrast to S-1 for which the maximum tolerated dose of 40 mg/m^2^/day has an associated 5-FU C_max_ of 128 ng/mL and 5-FU AUC of 724 ng·h/mL [24] and S-1 dosing at 30 mg/m^2^ BID has an associated 5-FU C_max_ of approximately 150 ng/mL and 5-FU AUC of approximately 800 ng·h/mL [50, 51]. S-1 doses above these MTDs were associated with Grade 3 or 4 gastrointestinal and myelosuppressive toxicities.

The PK characteristics of DFP-11207 continuous dosing indicate 5-FU concentration levels and 5-FU AUCs conducive to an anti-tumor effect and minimal toxicity, supported by preliminary evidence of anti-tumor activity suggest promise for future clinical trials of DFP-11207 in monotherapy or combination with standard chemotherapeutic drugs, specifically as a substitution for 5-FU, capecitabine or S-1 within standard 5-FU or oral 5-FU derivative treatment regimens for the treatment of a variety of 5-FU-responsive cancer indications.
